# Supplementary material for: Extra plasticity governed by shear band deflection in gradient metallic glasses
Source: Nat Commun. 2022 Apr 19;13:2120. doi: 10.1038/s41467-022-29821-4 (PMC9018681; doi:10.1038/s41467-022-29821-4)
Supplement: Supplementary file 1 — Supplementary Information [file 41467_2022_29821_MOESM1_ESM.pdf]

Supplementary Information for

Extra plasticity governed by shear band deflection in gradient metallic glasses

Yao Tang et al.

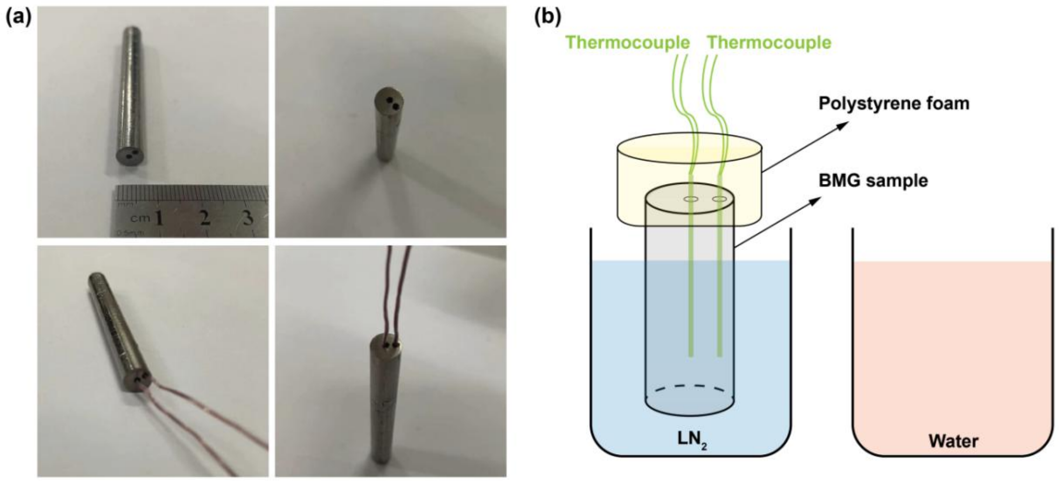

Supplementary Fig. 1 (a) Real photograph of the $Zr_{58}Cu_{22}Fe_8Al_{12}$ MG sample with two parallel holes. **(b)** Schematic diagram of temperature measurement tests using thermocouples.

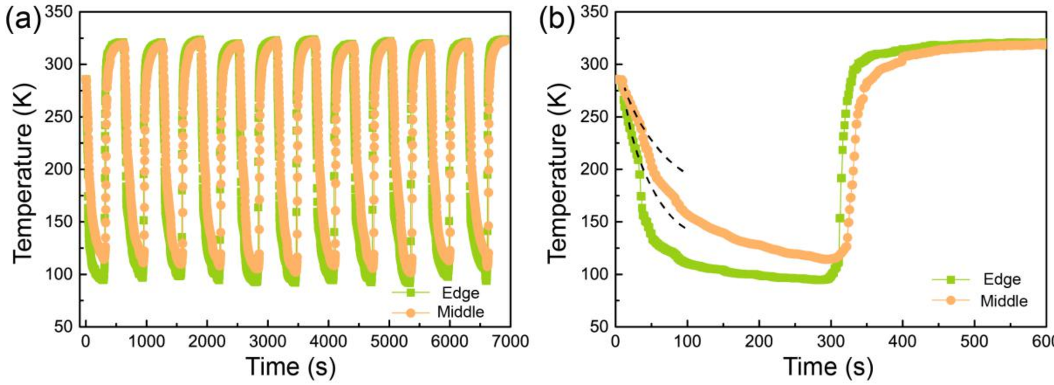

Supplementary Fig. 2 (a) The experimental time-temperature curves in the middle and edge of the $Zr_{58}Cu_{22}Fe_8Al_{12}$ MG sample with a diameter of 6 mm. **(b)** Enlarged view of one of the thermal cycles in **(a)**.

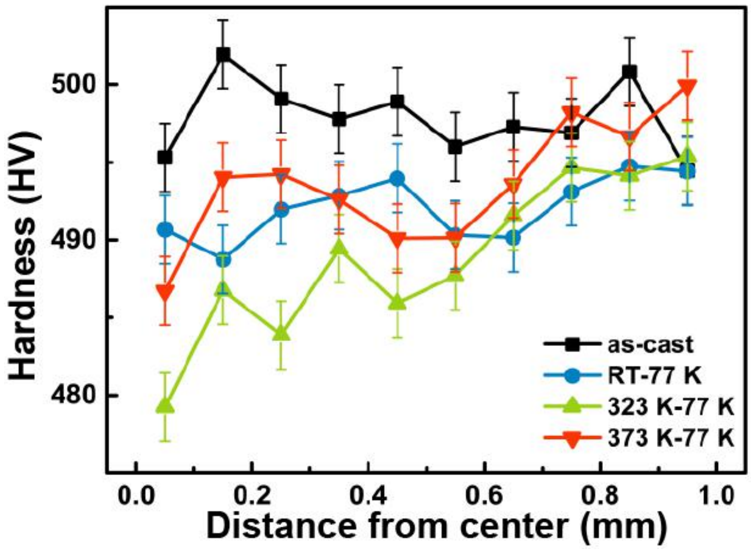

Supplementary Fig. 3 Variation of average hardness value along with the distance from the center in $Zr_{58}Cu_{22}Fe_8Al_{12}$ MGs by different CTC treatments in different temperature ranges. The error bars were obtained by standard deviation from fifteen independent measurements of hardness.

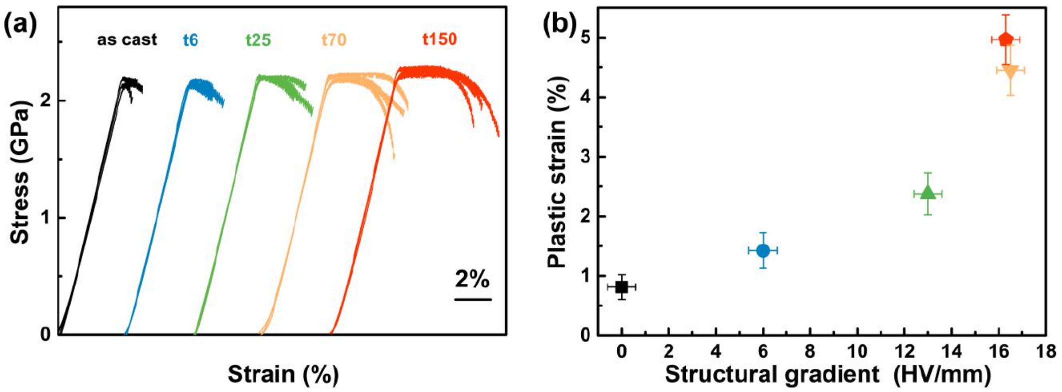

Supplementary Fig. 4 (a) Compressive stress-strain curves for the as-cast and treated $Zr_{58}Cu_{22}Fe_8Al_{12}$ MGs. **(b)** Variation of the plastic strain with the structural gradient for the as-cast and treated $Zr_{58}Cu_{22}Fe_8Al_{12}$ MGs. The vertical error bars represent standard deviation from four independent measurements of plastic strain. The horizontal error bars indicate the standard deviation of structural gradient according to the hardness value.

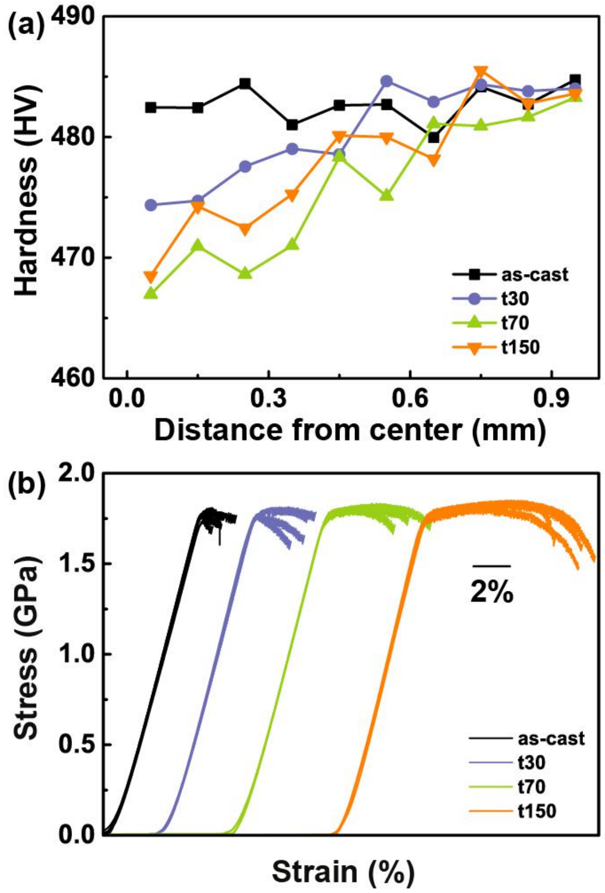

Supplementary Fig. 5 (a) Variation of average hardness value along with the distance from the center for as-cast and treated $Zr_{55}Cu_{30}Ni_{10}Al_5$ MGs. **(b)** Compressive stress-strain curves for as-cast and treated $Zr_{55}Cu_{30}Ni_{10}Al_5$ MGs.

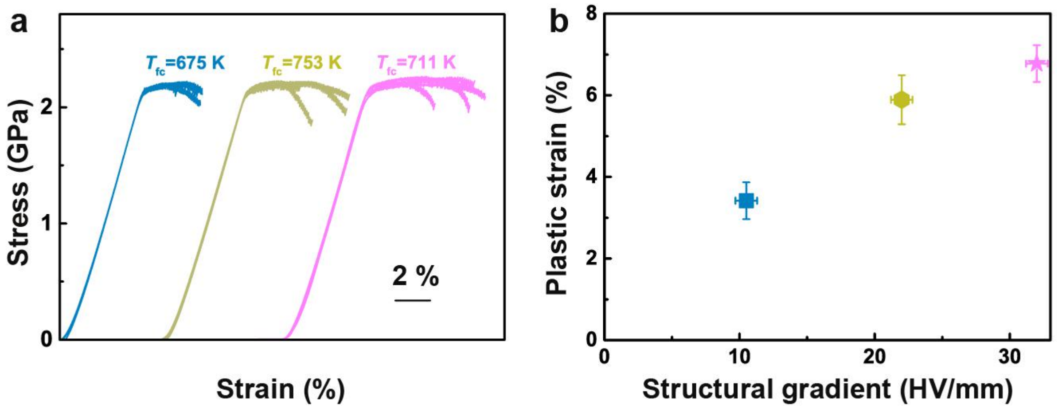

Supplementary Fig. 6 (a) Compressive stress-strain curves for the as-cast and treated $Zr_{58}Cu_{22}Fe_8Al_{12}$ MGs. **(b)** Variation of the plastic strain with the structural gradient for the as-cast and treated $Zr_{58}Cu_{22}Fe_8Al_{12}$ MGs. The error bars represent standard deviation from four independent measurements of plastic strain.

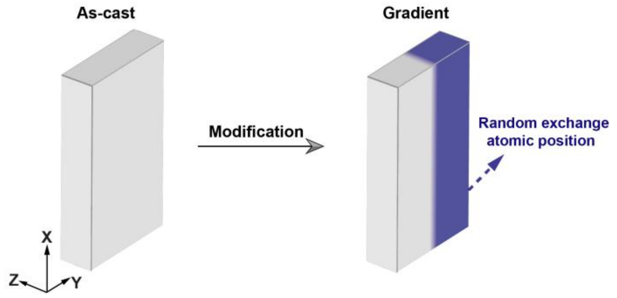

Supplementary Fig. 7 The schematic illustrations of the as-cast and gradient $Cu_{65}Zr_{35}$ MGs simulated by MD simulations.

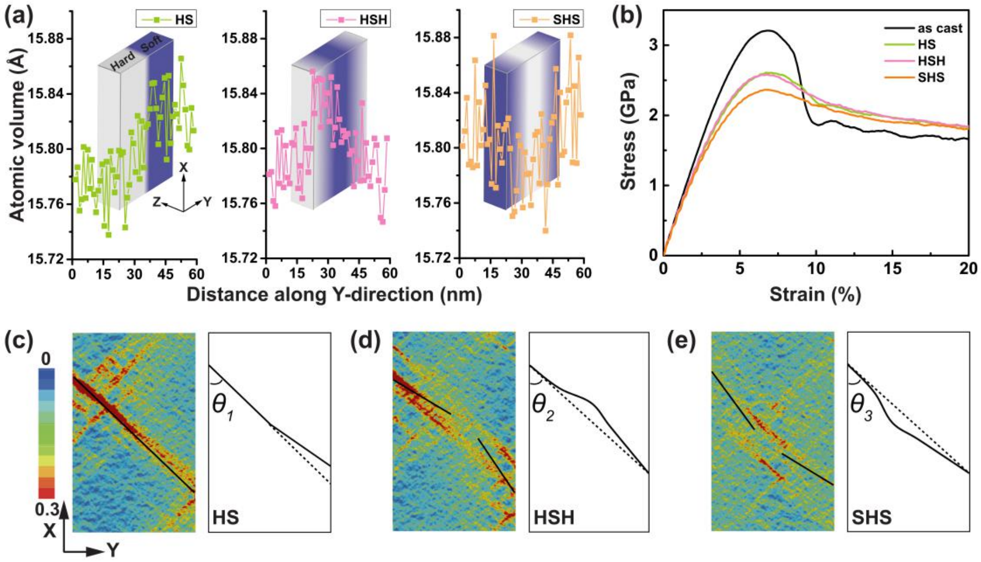

Supplementary Fig. 8 (a) Atomic free volumes as the functions of the position along the Y-direction in gradient $Cu_{65}Zr_{35}$ MGs simulated by MD simulations. **(b)** Representative stress-strain results for the as-cast and gradient $Cu_{65}Zr_{35}$ MGs simulated by MD simulations during compression along the X-direction. **(c-e)** The spatial distribution of atomic Mises strain of gradient $Cu_{65}Zr_{35}$ MGs simulated by MD simulations at 14% compression strain.

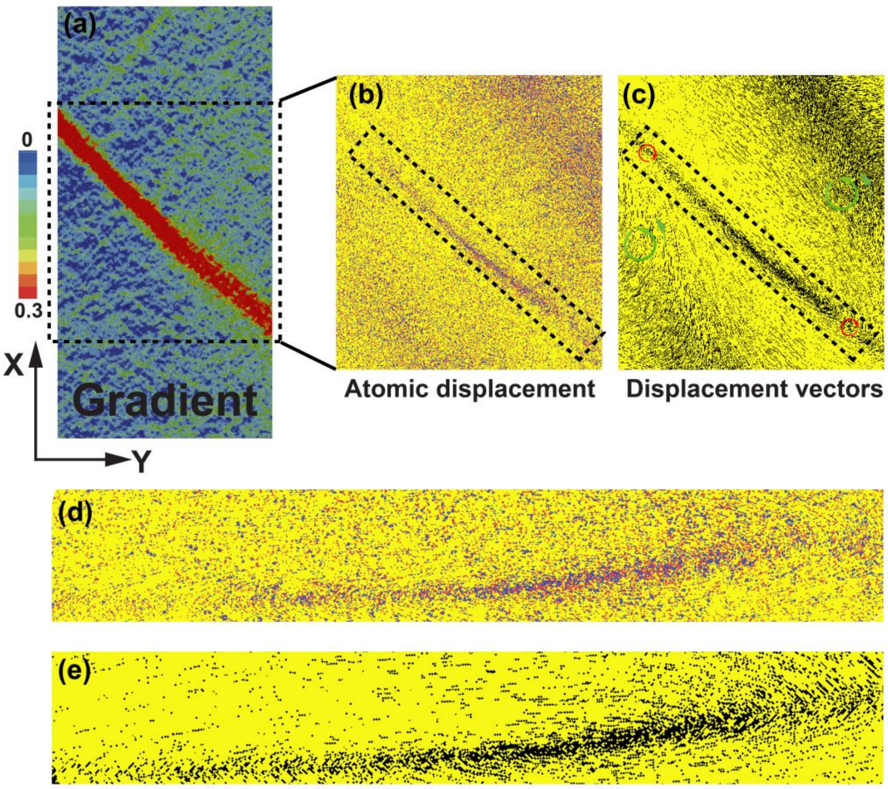

Supplementary Fig. 9 **(a)** Atomic Mises strain of the simulated gradient $Cu_{65}Zr_{35}$ MG (Hard-Soft) simulated by MD simulations. **(b)** Atomic displacement in the areas corresponding to the dotted boxes in **(a)**, the Cu atoms are shown in red and Zr atoms are shown in blue. **(c)** Representative vortex described by the displacement vectors. **(d)** An enlarged view of the dotted boxes in **(b)**, **(e)** An enlarged view of the dotted boxes in **(c)**.

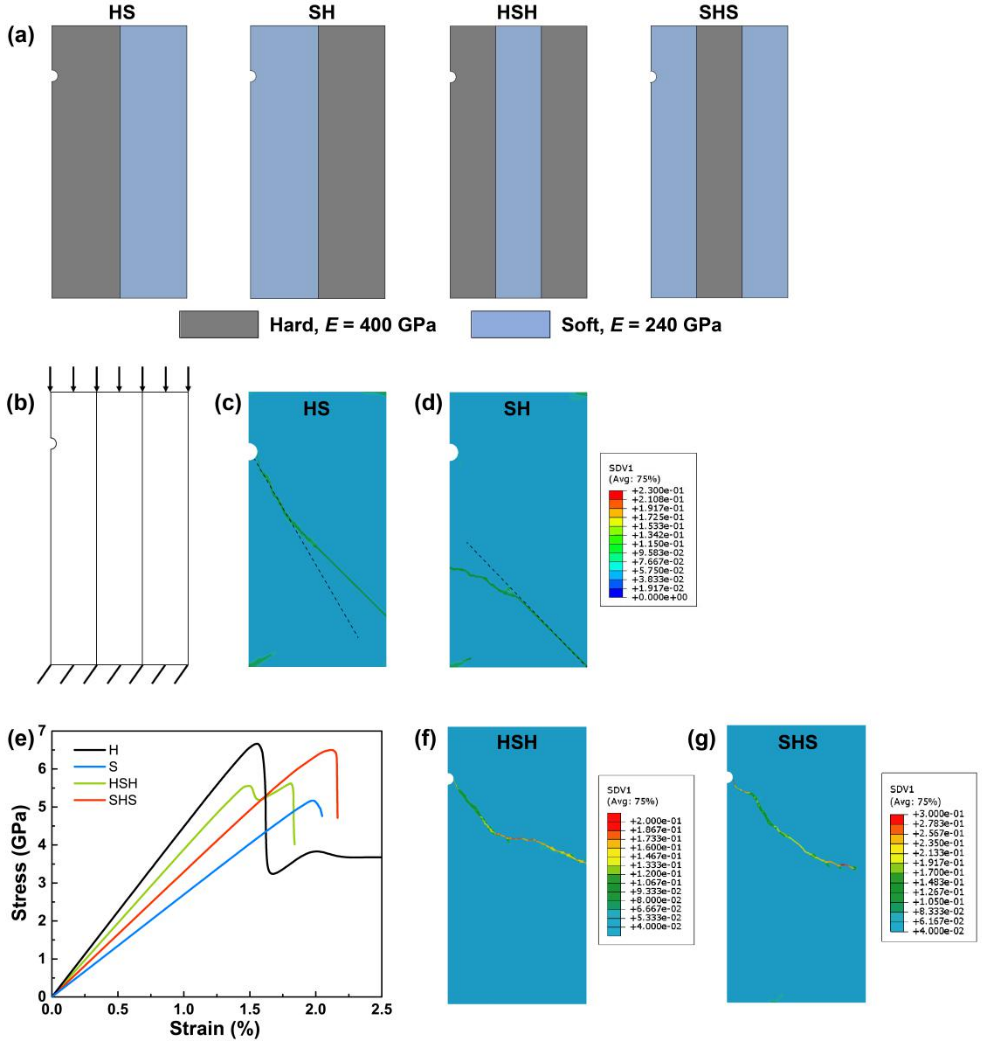

Supplementary Fig. 10 FEM simulations taking into account the change of modulus. (a) Different types of GMG models were produced by combining the hard (E = 400 GPa) and soft (E = 240 GPa) model. **(b)** Schematic diagram of compression of the notched model. **(c)** The shear band response of the HS MG model with deformation. **(d)** The shear band response of the SH MG model with deformation. **(e)** Comparison of the mechanical properties of HSH and SHS MG models with the pure hard and soft MG models. **(f)** and **(g)** show the shear band response of HSH and SHS MG models at maximum strain.

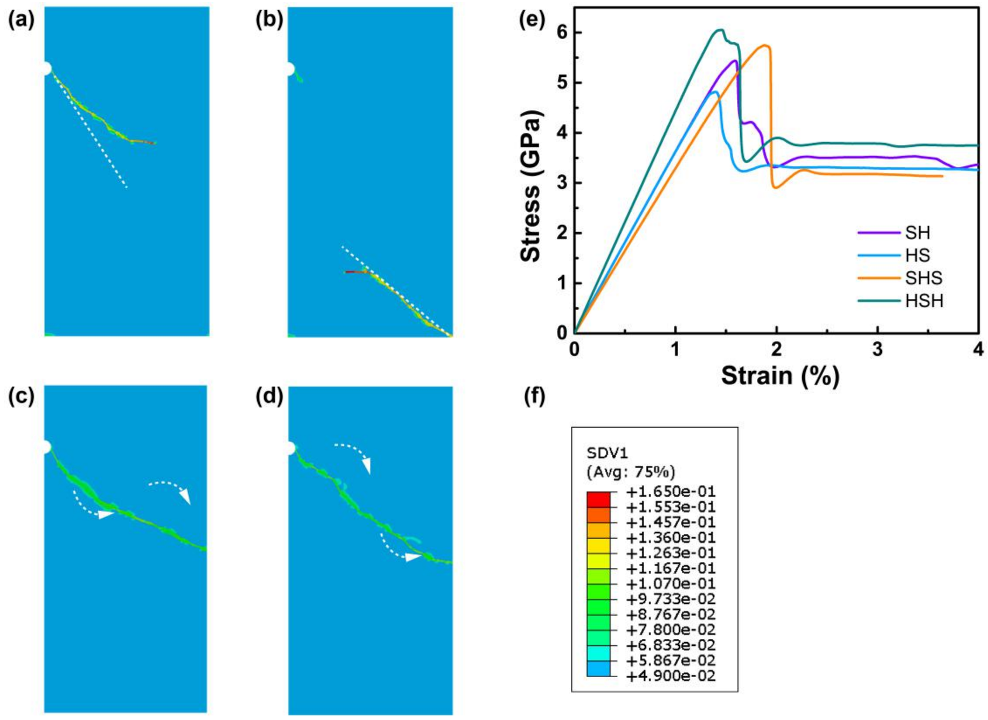

Supplementary Fig. 11 FEM simulations taking into account the change of free volume, Young's modulus and Poisson's ratio. (a) Shear band patterns for the Hard-Soft model. **(b)** Shear band patterns for the Soft-Hard model. **(c)** Shear band patterns for the Soft-Hard-Soft model. **(d)** Shear band patterns for the Hard-Soft-Hard model. **(e)** Stress-Strain curves for various GMG models. **(f)** V_f scale bar for **(a-d)**.

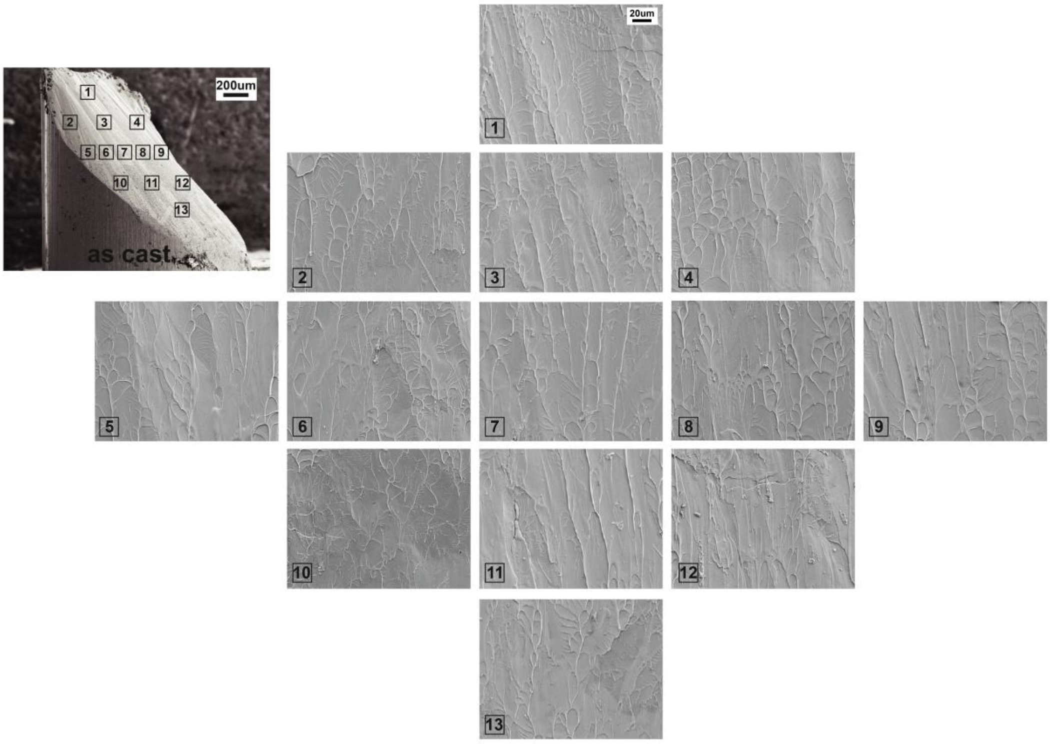

Supplementary Fig. 12 SEM surface morphologies at different positions in fractured as-cast $Zr_{58}Cu_{22}Fe_8Al_{12}$ sample.

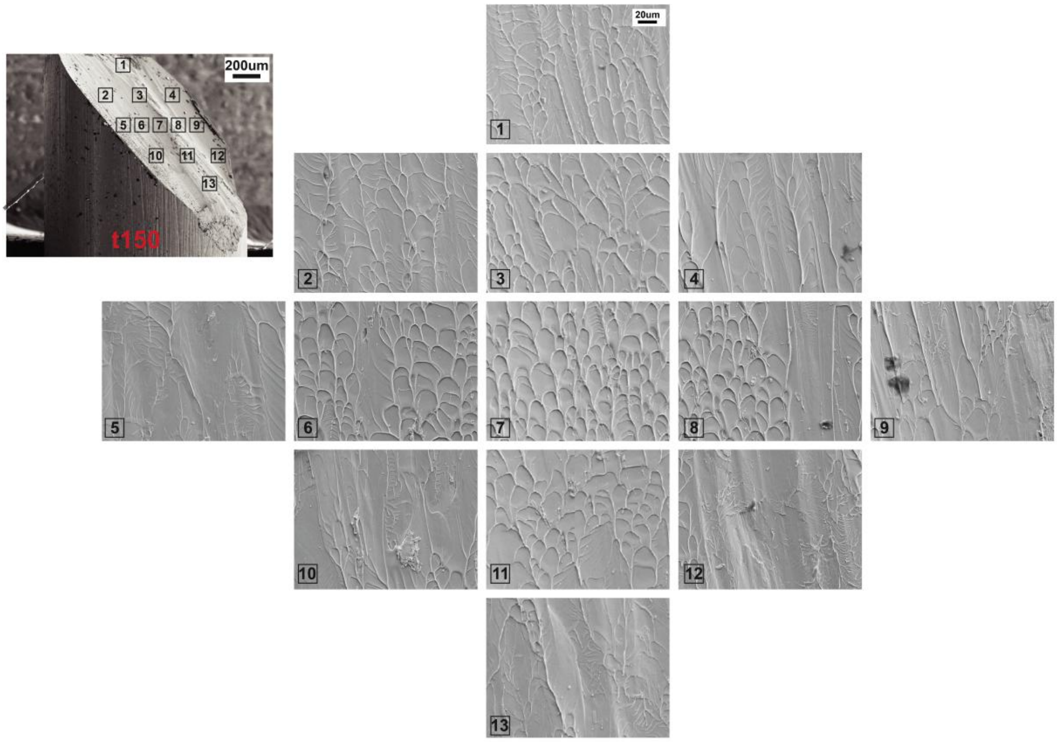

Supplementary Fig. 13 SEM surface morphologies at different positions in fractured t150 $Zr_{58}Cu_{22}Fe_{8}Al_{12}$ sample.

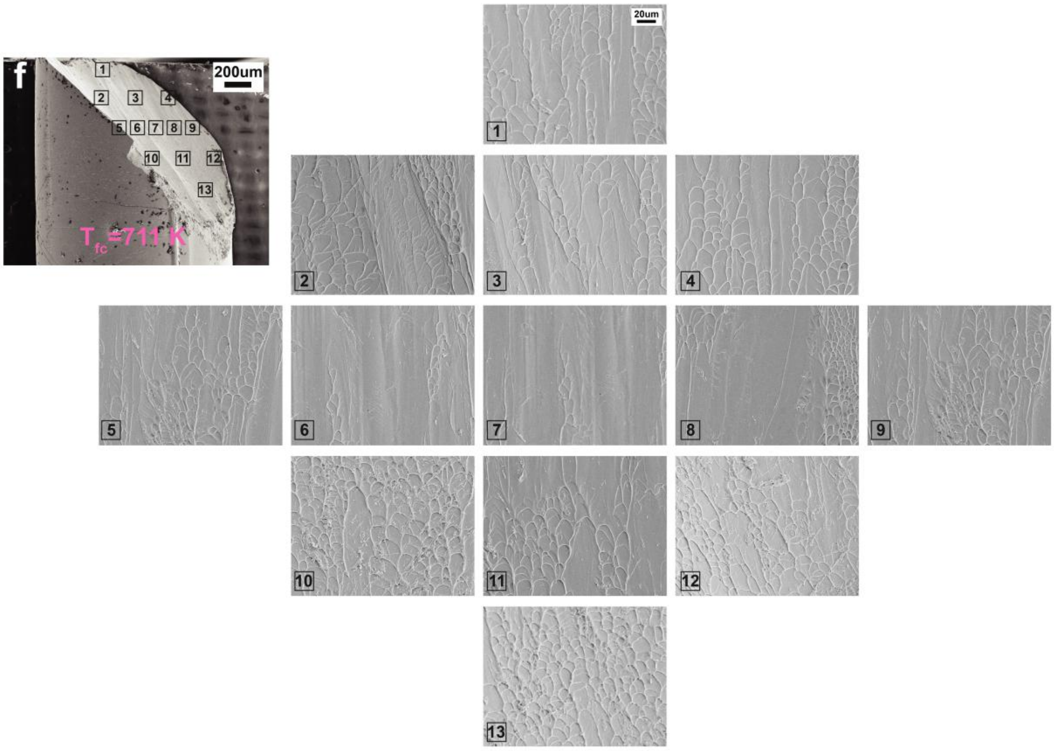

Supplementary Fig. 14 SEM surface morphologies at different positions in fractured T_{fc}=711 K $Zr_{58}Cu_{22}Fe_{8}Al_{12}$ sample.

Supplementary Note 1 | Heat transfer in LN_2 environment. The Biot number (Bi) is defined as Bi = *hl/k*, where *k* is the thermal conductivity, *h* is the heat transfer coefficient, and *l* is the characteristic size of the sample. In our work, *l* is the characteristic length scale equal to the radius of the cylindrical rod sample. In our calculations, we use material data for the typical ZrCu-based BMG. We choose 4 W m^{-1} K^{-1} for *k* in the heating process and 2 W m^{-1} K^{-1} for *k* in the cooling process. We lack data on the exact values of the experimental heat-transfer coefficients typically found in BMG processing. To obtain a rough estimate, we note that in our cryogenic thermal cycling (CTC) process, BMG samples are heated in hot water (T = 323 K) and cooled in LN_2. The convection heat-transfer coefficient for free convection of water at room temperature is about 900 W $(m^2{\cdot}K)^{-1}$. If the water boils, then values up to 35000 W $(m^2{\cdot}K)^{-1}$ are possible[1]. In the cooling process corresponding to the film boiling regime, the heat-transfer coefficient for LN_2 is limited (<1000 W $(m^2{\cdot}K)^{-1}$[2]) and for nucleate-boiling h = 1355 ± 51 W $(m^2{\cdot}K)^{-1}$[3]. To be conservative, we chose 5000 W $(m^2{\cdot}K)^{-1}$ for *h* in the heating process and 1400 W $(m^2{\cdot}K)^{-1}$ for *h* in the cooling process. According to the definition, we calculated the Biot number values in hot water (T = 323 K) and LN_2 (T = 77 K), as presented in Supplementary Table 1. For a 2 mm diameter cylindrical sample, the corresponding Bi value is about 2.5 in hot water (T = 323 K) and 0.7 in LN_2 (T = 77 K). In the present study, the Biot number is on the order of 1, which means that the thermal conduction within the BMG during cooling in the LN_2 environment cannot be neglected and the heat transfer is rate controlling.

In addition to the Biot number, when dealing with liquid nitrogen (LN_2), the temperature difference between the fluid and the sample is large enough to cause boiling of the liquid entering into the film boiling regime[4,5]. This determines a heat flux from the object to LN_2, creating a pocket of nitrogen vapor around the solid which acts as an 'insulator' retarding further heat transfer. The object will cool down, rather slowly due to the low heat transfer rates during film boiling. Vapor film will then break off while the heat flux progressively increases as transition to the nucleate boiling regime is established[6]. This event is characterized by a steep increase in heat flux. Therefore, because of the existence of film boiling and nucleate boiling, it will take a long period for heat transfer during liquid nitrogen cooling. Our new temperature measurement results in LN_2 (Supplementary Fig. 1 and Fig. 2)

also show the existence of a two-stage boiling regime phenomenon.

CTC Medium	Heat Transfer *h*	Characteristic size *l*	Thermal conductivity *k*	Biot Number
Hot water	5000 W $(m^2 \cdot K)^{-1}$	1 mm	4 W m^{-1} K^{-1}	1.25
Liquid nitrogen	1400 W $(m^2 \cdot K)^{-1}$	1 mm	2 W m^{-1} K^{-1}	0.7

Supplementary Table 1. Material parameters and Biot number of ZrCu-based BMG in different media.

Supplementary Note 2 | Important factors (holding time and temperature) affecting the outcome of thermal cycling. In the previous work[7], the CTC method is to hold one minute at both room temperature and liquid nitrogen temperature. For the previous way, the cryogenic thermal treatment may not affect the relaxation of MGs because of the low atomic mobility at room temperature. Previous work did not adjust the CTC parameters and capture the details of the gradient distributed free volume. In addition, previous work did not provide direct experimental evidence to measure or calculate the temperature distribution in the cooling or heating sample. We ascertained this information by conducting additional experiments to show changes in internal and external temperature of MG samples during thermal cycling. Considering the diameter of the thermocouple and the necessity to place the thermocouple in the body of the sample, we therefore used a cylindrical $Zr_{58}Cu_{22}Fe_{8}Al_{12}$ MG sample with a diameter of 6 mm in the temperature measurement. Note that the $Zr_{58}Cu_{22}Fe_{8}Al_{12}$ MG has a good glass-forming ability and can be cast into a fully amorphous rod with a diameter of up to 13 mm[8]. We drilled two parallel holes in the middle and edge of the sample, and then placed a thermocouple in each hole to measure the local temperature. The photograph of the actual sample is shown in Supplementary Fig. 1a. In Supplementary Fig. 1b, the schematic diagram of temperature measurement tests is shown. The depth of the holes is large enough to ensure that the position of the thermocouples is far below the LN_2 surface. To prevent liquid nitrogen from splashing into the holes, the upper part of the BMG sample was enclosed by a polystyrene foam. The time-temperature curve was recorded using type T thermocouples (Copper-Constantan, especially used to measure low temperatures).

Supplementary Fig. 2 shows the experimental results of our temperature measurement at two holes during cryogenic thermal cycling with a holding time of 300 s. For better comparison, we enlarged one of the thermal cycles in Supplementary Fig. 2a. As can be seen in Supplementary Fig. 2b, the temperature of the two holes decreases linearly. Afterwards, an abrupt slope change in the cooling curve develops and a rapid drop in the temperature is observed which corresponds to the transition of film to nucleate boiling. The hole in the edge of the sample reached the liquid nitrogen temperature faster than that the hole in the middle of the sample. After 300 s, the temperature of the hole in the middle was still lower than that of the hole in the edge. During the heating process, the hole in the edge of the sample also

reached 323 K faster than that the hole in the middle of the sample. The temperature of the hole in the middle was almost the same as that of the hole in the edge after 600 s. The observations demonstrate that the heat transfer of the BMG sample at liquid nitrogen temperature is very different from that at hot water. This is mainly due to the difference of the Biot number at different environments and the film/nucleate boiling phenomenon when dealing with cryogenic LN_2.

Another important point we also want to emphasize is that temperature is also an important factor affecting the outcome of thermal cycling. The normal CTC method to rejuvenate MGs is to hold one minute at both room temperature and liquid nitrogen temperature. To maximum the rejuvenation effect, we used the 323 K-77 K. For comparison, we also performed the CTC treatment at 373 K-77 K. Supplementary Fig. 3 shows the results of these three CTC treatments in different temperature ranges. It can be seen that the hardness value has no obvious gradient change when cycling at RT-77 K. The rejuvenation effect in the center of the sample appears to be more pronounced when cycling at 323 K-77 K. However, when cycling at 373 K-77 K, the hardness value increases relative to that when cycling at 323 K-77 K. This is because the atoms will move cooperatively and result in the relaxation when a long holding time at high temperature. These results prove again that the importance of the processing conditions. By adjusting these processing conditions, the gradient structure could be produced and maximum rejuvenation could be achieved.

Supplementary Note 3 | The effect of water on the surface of the sample for the CTC experiments. We designed a machine to automatically carry out the CTC experiments in hot water and liquid nitrogen. It took about 6 s for the sample to be taken out from hot water and transferred to liquid nitrogen. There is little water left on the surface of the sample before it enters liquid nitrogen. Water has limited effect on the temperature transfer on the surface of the sample. As can be seen in Supplementary Fig. 2, the surface of the sample quickly reached the liquid nitrogen temperature within a few seconds. The gradual changes of the hardness (Supplementary Fig. 3) suggest that the generation of the gradient is mainly related to the holding time. After the CTC experiments, we polished the surface of the treated MG samples before doing subsequent measurements.

Supplementary Note 4 | Universality of cryogenic thermal cycling method. To verify that our method is not only specific for this system, we have also performed the same heat treatment engineering protocol to realize a controllable structural gradient in $Zr_{55}Cu_{30}Ni_{10}Al_5$ MG. Supplementary Fig. 5 shows the variation of hardness across the diameter on a cross-section of the 2 mm cylindrical as-cast and treated $Zr_{55}Cu_{30}Ni_{10}Al_5$ samples. Notably, a gradient of the hardness value can be detected for the t30 sample. In particular, a more obvious hardness-value gradient can be seen for the t70 and t150 samples. To demonstrate the enhanced plastic deformability of gradient BMGs, we compared the engineering compressive stress-strain curves of the gradient $Zr_{55}Cu_{30}Ni_{10}Al_5$ samples with the as-cast sample (Supplementary Fig. 5b). It can be seen that the plasticity strongly increased significantly without the expense to the strength in the gradient $Zr_{55}Cu_{30}Ni_{10}Al_5$ samples. These results verified that this process can be implemented for other metallic glass systems.

Supplementary Note 5 | Another 'accelerate' method to construct the GMG in MD simulations. In this work, MD simulations were performed to artificially construct the gradient MG, and to explore the mechanism of shear band deflection. Because of the time scale[9-11], the temperature transfer of the sample is instantaneous in MD simulations. The formation of the structural gradient cannot be explored by MD simulations, and it is impossible to produce GMG in such a short time scale. Therefore, we can only artificially construct GMG by the 'accelerate' method. In the manuscript, we constructed GMG by randomly removing the atoms, but the composition of the sample was unchanged.

Now, we have performed another 'accelerate' method without changing the number of atoms to construct the GMG in MD simulation. As shown in Supplementary Fig. 7, 10% of Zr atoms were randomly selected and replaced with Cu atoms in the right half of the box. Then, 5.11% of Cu atoms were also randomly chosen and set as Zr atoms in the right half of the box. The average atomic volume will increase in the atomic exchange regions. In fact, this 'exchange atom' method is very close to the random change of free volume during thermal cycling. Three types of gradient MGs fabricated with different atomic exchange regions are used to explore the characteristics of shear band deflection, as an illustration in Supplementary Fig. 8. The HSH (Hard-Soft-Hard) MG and SHS (Soft-Hard-Soft) MG correspond to the two GMGs with different structures in experiments. The propagation of the shear band appeared to be changed when it penetrated to the soft region in the HS MG (Supplementary Fig. 8c). For the HSH MG (Supplementary Fig. 8d), the primary shear band initiates at the surface with a relatively lower content of free volumes, as the shear band progresses toward the central soft region of the specimen, the increasing value of free volume concentration will alter the normal stress effect on the shear band, inducing a gradual increase in the shear band angle. For the SHS MG (Supplementary Fig. 8e), the primary shear band initiates at the center with a relatively lower content of free volumes, it can be expected that as the shear band propagates from the center to the surface, free volume concentration declines, leading to a gradual increase in the shear band angle. The humps and concavities indicate different shear band behaviors for the two GMGs, which are very consistent with our experimental observations. The above evidence suggests that this 'exchange atom' method can be also applied to construct the GMG and allow us to quantitatively capture the

fundamental characteristics of shear band deflection in GMG. Like the ‘remove atom’ method, they do not change the composition of the sample.

Supplementary Note 6 | Atomic displacement vectors of the GMG (Hard-Soft) simulated by MD simulations. Supplementary Fig. 9b shows the atomic displacement vectors corresponding to the dotted boxes in Supplementary Fig. 9a. It can be seen that the circular displacements on the left and right sides of the sample are very different. Because there are too many atoms, a plate with a thickness of 10 nm was taken from the sample to directly display the displacement vectors without atoms, as shown in Supplementary Fig. 9c. The atomic displacement vectors in the shear band reveal the presence of regions where atoms describe circular displacements resembling a collective, clockwise vortexlike motion. According to the displacement vectors around shear bands in the left regions, the representative anti-clockwise vortexlike motion is considered. These anti-clockwise vortex-like motions can act as a medium, triggering the activation of clockwise vortexlike motion (red color), and cause the rapid propagation of the shear band. However, the atomic displacement vectors around shear bands change in the right soft region. The displacement vectors below the shear bands become less, while the displacement vectors above the shear bands become more obvious. The representative anti-clockwise vortexlike motion above the shear bands will govern the clockwise vortexlike motion (red color) to the upper right direction. Supplementary Figs. 9d and e show the enlarged views of the dotted boxes in (b) and (e). It can be seen that the vortex field deflects upward to the right, and the angle of the STZ percolation path changes to a larger value than that in the left region. The results of atomic displacement vectors can support the explanation of the STZ percolation mechanism in gradient metallic glass.

Supplementary Note 7 | Finite element modeling on shear band behavior in GMGs. To better illuminate the behaviors of shear band propagation in the gradient MGs, we also performed the Finite element method (FEM) analysis (Supplementary Fig. 10). Although the GMG is also constructed artificially, the FEM does not involve the problem of the potential function, and the strain rate is also relatively low. For simplicity, the actual three-dimensional deformation conditions were reduced to two-dimensional plane-strain models, for uniaxial compression. Meanwhile, the shear band formation model[12] proposed by Y.F. Gao was adapted to approximate the deformation behavior of the MGs. The models were discretized with 40000 elements using the plane-strain three-node triangular element. The ratio of the height of the models and its width is kept at 2, which is the same as the models used in our MD simulations. Different types of GMGs were produced by combining the hard (E = 400 GPa) and soft (E = 240 GPa), as shown in Supplementary Fig. 10a. To control the shear band initiation site, a small notch was installed on the left surface, acting as a stress concentrator. The bottom of the models is fixed, and a strain rate of 5×10^{-6} is applied to compress the model. For the HS (Hard-Soft) MG model, the shear band propagates from the left hard region and deflects in the right soft region. For the SH (Soft-Hard) MG model, the shear band also propagates from the right hard region and deflects in the left soft region. These results suggest that shear band deflection indeed occurs during deformation in GMGs. We also used HSH and SHS MG models to observe the different shear band behaviors in different GMGs. We have compared the mechanical properties of HSH and SHS MG models with the pure hard and soft MG models. As shown in Supplementary Fig. 10e, the moduli of HSH and SHS MG models are indeed between that of pure hard and soft MG. The patterns of the shear band can be seen in Supplementary Fig. 10f and g. For the HSH MG model, as the shear band propagates toward the central soft region, it shows a significant upward deflection. As the shear band propagates from the center soft region to the right hard region, a reversed deflection pattern can be observed. For the SHS MG model, an obvious concavity can be observed in the middle.

To be more based on the experimental facts of the sample, we have carried out additional FEM simulations by taking into account the change of free volume. The coupling

effects of free volume V_f, Young's modulus E and Poisson's ratio ν are considered in the additional FEM simulations. The free volume V_f is a normalized value that has been clarified by Gao[12]:

$$V_f = \frac{V_f^*}{\alpha V} \tag{1}$$

where V_f^* is the actual free volume value of one atom and V is the total value of the atom. $\alpha = 0.15$ is used here, which is the same as that used in Gao's work. E = 240 GPa and E = 400 GPa, $V_f = 0.052$ and $V_f = 0.05$, $\nu = 0.333$ and $\nu = 0.35$ are chosen for the soft part and the hard part, respectively. A 0.2% change in V_f corresponds to a 1.33% change in the actual free volume V_f^*, which is on the same order to those in our experiments. As mentioned by previous work[13,14], with the decrease of the free volume, E and ν will increase. Considering that these parameters are coupled together, we have taken these factors into account to construct four different types of GMGs, as shown in Supplementary Fig. 11. The samples size and loading methods remain unchanged as the previous FEM simulation. The shear band patterns and mechanical properties of the GMG models are presented in Supplementary Fig. 11. These results reveal different shear band behaviors related to the gradient-distributed free volume contents and are in good agreement with the FEM simulation results shown in Supplementary Fig. 10. These results validate the two novel shear band behaviors related to the gradient-distributed free volume contents and are in good agreement with our experimental observations. Our finite element simulations convincingly demonstrate that gradient structure can induce shear band deflection in MGs.

Supplementary References

1. Holman, J. P. : Heat Transfer, 6th ed., McGraw-Hill, New York, NY, 1986, p. 13.

2. Han, X., Ma, H. B., Jiao, A., Critser, J. K. Investigations on the heat transport capability of a cryogenic oscillating heat pipe and its application in achieving ultra-fast cooling rates for cell vitrification cryopreservation. *Cryobiology* **56**, 195-203 (2008).

3. Santos, M. V., Sansinena, M., Chirife, J., Zaritzky, N. Determination of heat transfer coeffificients in plastic French straws plunged in liquid nitrogen. *Cryobiology* **69**, 488-495 (2014).

4. Bui, T. D., Dhir, V. K. Film boiling heat transfer on an isothermal vertical surface. J. *Heat Transfer Trans ASME* **107**, 764-771 (1985).

5. Suryanarayana, N. V., Merte, Jr. H. Film boiling on vertical surfaces. *J. Heat Transfer Trans ASME* **94**, 377-384 (1972).

6. Hsu, Y. Y. NASA TM TECHNICAL PAPER Cryogenic Engineering Conference, 1970.

7. Ketov, S. V., Sun, Y. H., Nachum, S., Lu, Z., Checchi, A., Beraldin, A. R., Bai, H. Y., Wang, W. H., Louzguine-Luzgin, D. V., Carpenter, M. A., Greer, A. L. Rejuvenation of metallic glasses by non-affine thermal strain. *Nature* **524**, 200-203 (2015).

8. Jin, K. F., Löffler, J. F. Bulk metallic glass formation in Zr-Cu-Fe-Al alloys. *Appl. Phys. Lett.* **86**, 241909 (2005).

9. Schiotz, J., Tolla, F., Jacobsen, K. W. Softening of nanocrystalline metals at very small grain sizes. *Nature* **391**, 561-563 (1998).

10. Yamakov, V., Wolf, D., Phillpot, S. R., Mukherjee, A. K., Gleiter, H. Dislocation processes in the deformation of nanocrystalline aluminium by molecular-dynamics simulation. *Nat. Mater.* **1**, 45-49 (2002).

11. Zhang, P., Ma, L. L., Fan, F. F., Zeng, Z., Peng, C., Loya, P. E., Liu, Z., Gong, Y. J., Zhang, J. N., Zhang, X. X., Ajayan, P. M., Zhu, T., Lou, J. Fracture toughness of graphene. *Nat. Commun.* **5**, 3782 (2014).

12. Gao, Y. F. An implicit finite element method for simulating inhomogeneous deformation and shear bands of amorphous alloys based on the free-volume model. *Model. Simul. Mater. Sc.* **14**, 1329-1345 (2006).

13. Wang, W. H., Li, F. Y., Pan, M. X., Zhao, D. Q., Wang, R. J. Elastic property and its response to pressure in a typical bulk metallic glass. *Acta Mater.* **52**, 715-719 (2004).

14. Caris, J., Lewandowski, J. J. Pressure effects on metallic glasses. *Acta Mater.* **58**, 1026-1036 (2010).
